# Supplementary material for: Taxifolin Inhibits the Growth of Non-Small-Cell Lung Cancer via Downregulating Genes Displaying Novel and Robust Associations with Immune Evasion Factors
Source: Cancers (Basel). 2023 Sep 30;15(19):4818. doi: 10.3390/cancers15194818 (PMC10571863; doi:10.3390/cancers15194818)
Supplement: Supplementary file 1 [file cancers-15-04818-s001.zip › Sup Figs S1-S12.pdf]

Figure S1

A

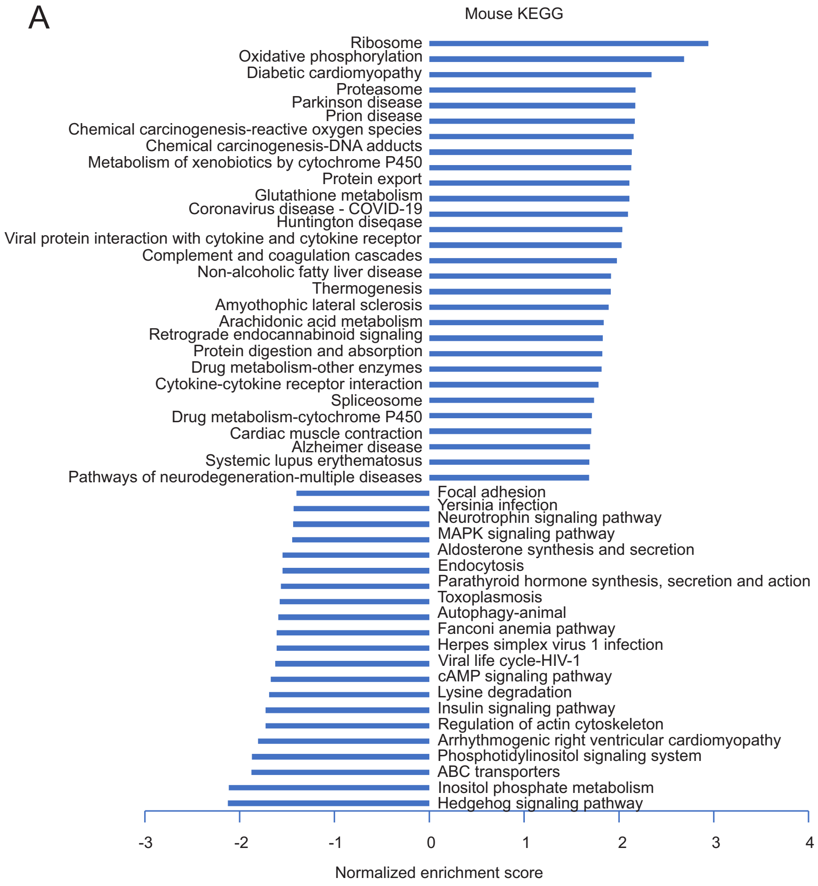

B

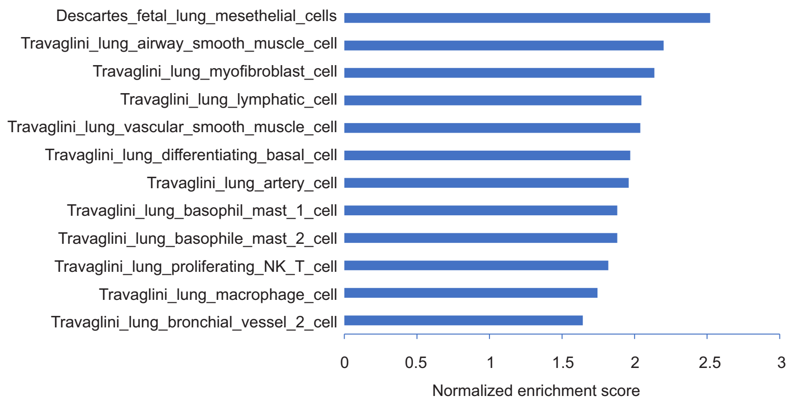

Figure S1. Taxifolin-mediated enrichment of pathway in LL2 tumors. (**A**) GSEA analysis of gene expression in LL2 tumors affected by taxifolin in comparison to DMSO-treated LL2 tumors using KEGG gene set. Analysis was performed using ClusterProfiler. (**B**) The human homologous genes of the above LL2 tumors were analyzed by GSEA using MSigDB C8 cell type signature gene sets.

Figure S2

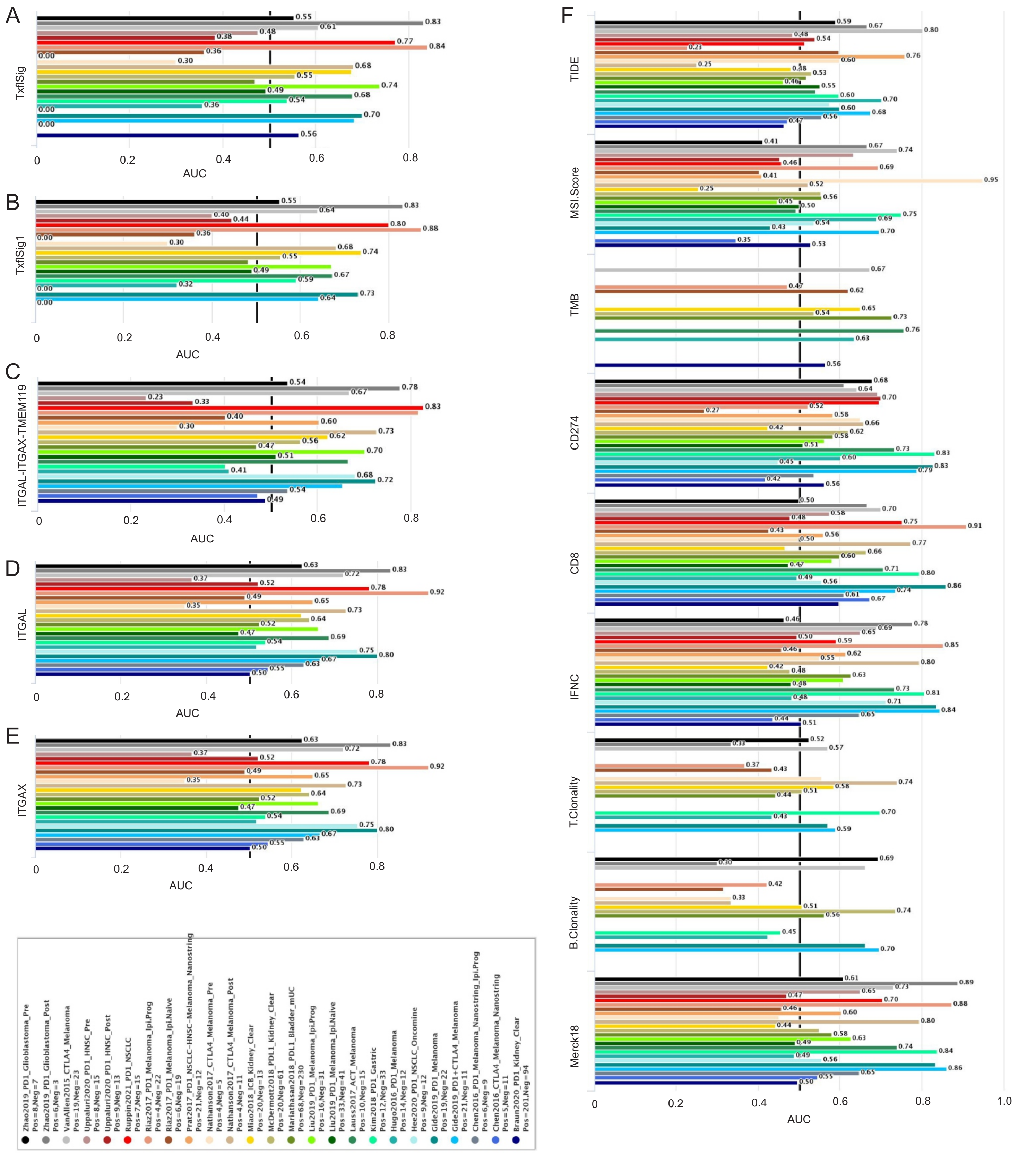

Figure S2. Prediction of resistance to ICB. Evaluation of TxflSig, TxflSig, IIT (ITGAL+ITGAX+TMEM119), ITGAL, and ITGAX (A-E) along with a set of published ICB biomarkers (F) in the set of ICB-treated cohorts organized by the TIDE platform (http://tide.dfci.harvard.edu/login/).

Figure S3

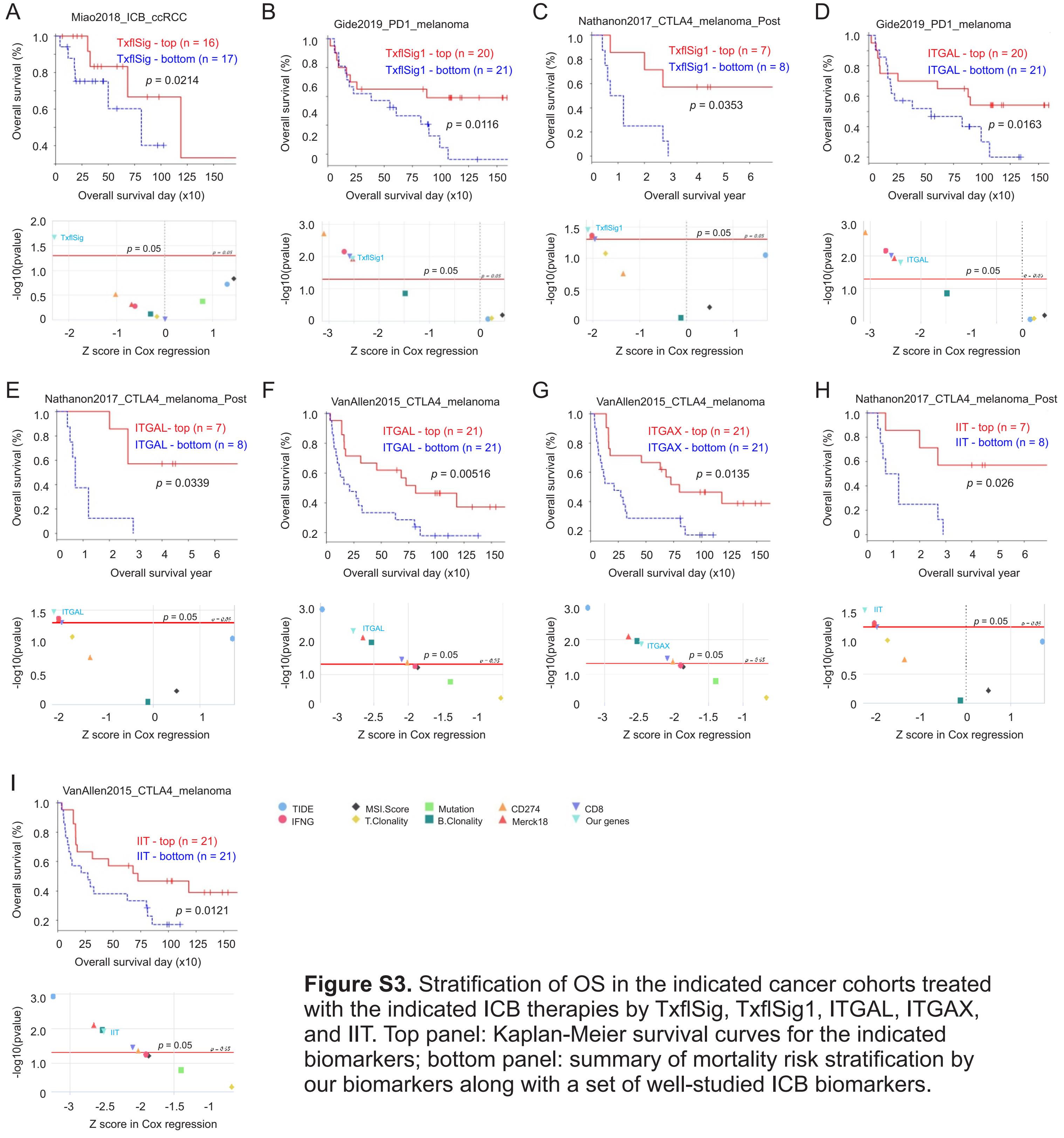

Figure S3. Stratification of OS in the indicated cancer cohorts treated with the indicated ICB therapies by TxflSig, TxflSig1, ITGAL, ITGAX, and IIT. Top panel: Kaplan-Meier survival curves for the indicated biomarkers; bottom panel: summary of mortality risk stratification by our biomarkers along with a set of well-studied ICB biomarkers.

Figure S4

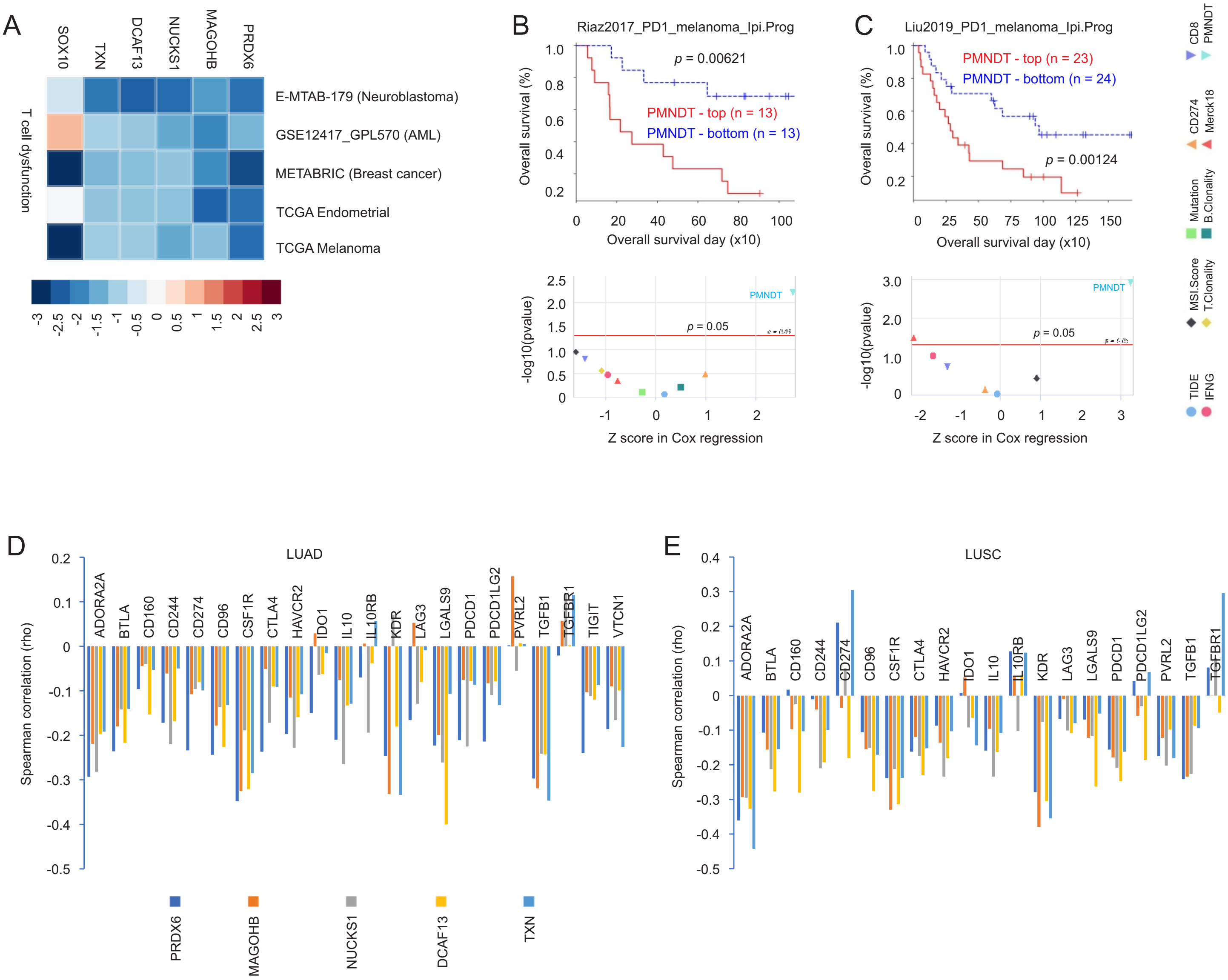

Figure S4. Negative association of taxifolin-upregulated genes with factors promoting immune evasion in NSCLC. (**A**) Association of the indicated DEGs upregulated by taxifolin in LL2 tumors with T cell dysfunction value; analysis was performed using the TIDE platform. (**B, C**) PRDX6, MAGOHB, NUCKS1, DCAF13, and TXN were analyzed as a panel (PMNDT) in the stratification of OS probability in the indicated cohort. Analysis was performed using the TIDE platform. (**D, E**) Negative correlation of PRDX6, MAGOHB, NUCKS1, DCAF13, and TXN with the indicated immune checkpoints in LUAD and LUSC. Correlation (spearman) analysis was performed using the TISIDB platform.

Figure S5

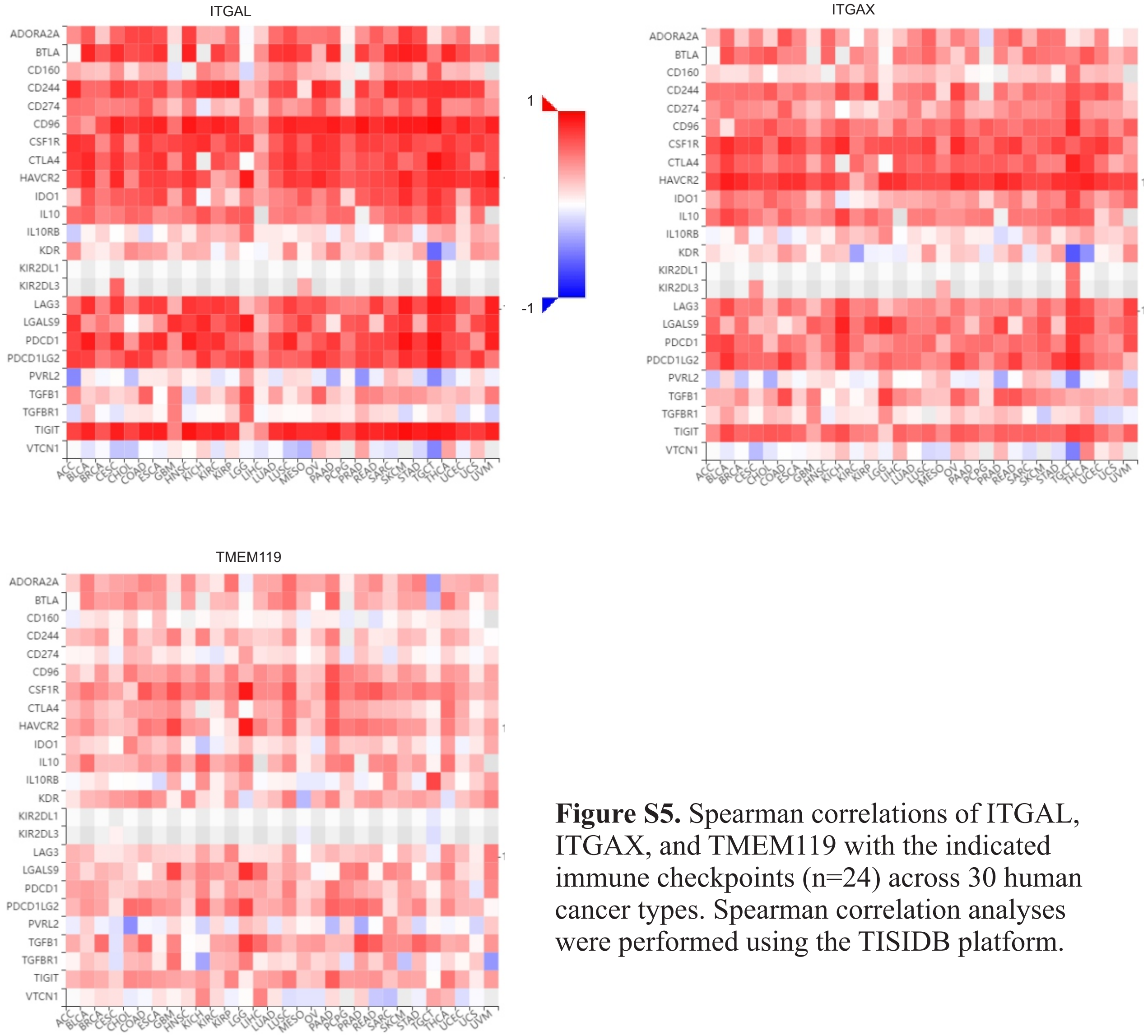

Figure S5. Spearman correlations of ITGAL, ITGAX, and TMEM119 with the indicated immune checkpoints (n=24) across 30 human cancer types. Spearman correlation analyses were performed using the TISIDB platform.

Figure S6

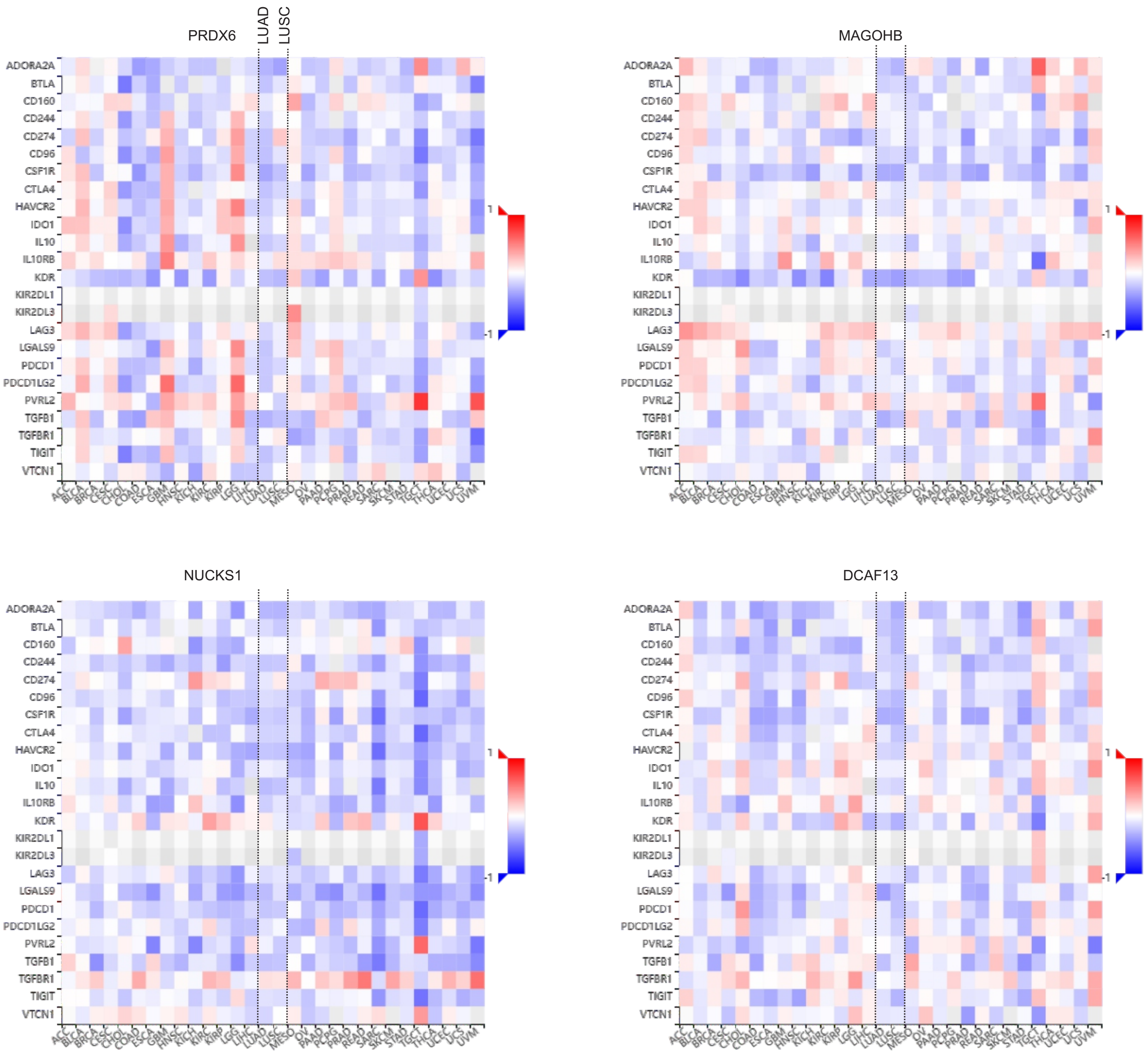

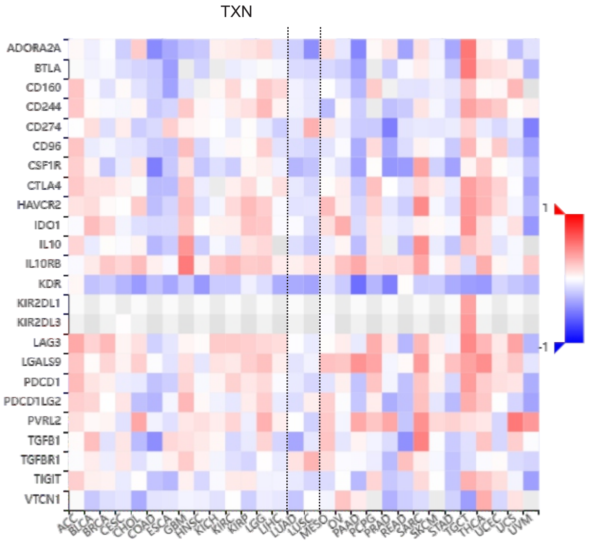

Figure S6. Spearman correlations of the indicated genes with immune checkpoints. PRDX6, MAGOHB, NUCKS1, DCAF13, and TXN, whose mouse homologous genes were upregulated by taxifolin in LL2 tumors, were analyzed for spearman correlation with the indicated immune checkpoints (n = 24) across 30 human cancer types using the TISIDB platform. Both LUAD and LUSC cancer types are bordered by dotted lines. Please note the general negative correlations of these genes with the indicated immune checkpoints in LUAD and LUSC.

Figure S7

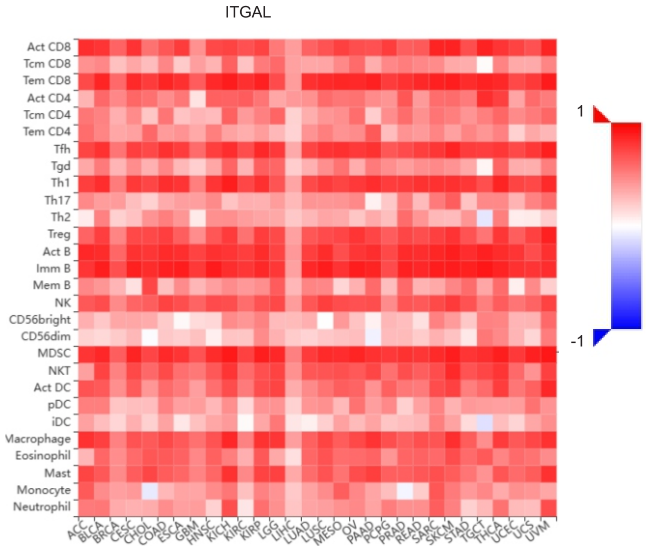

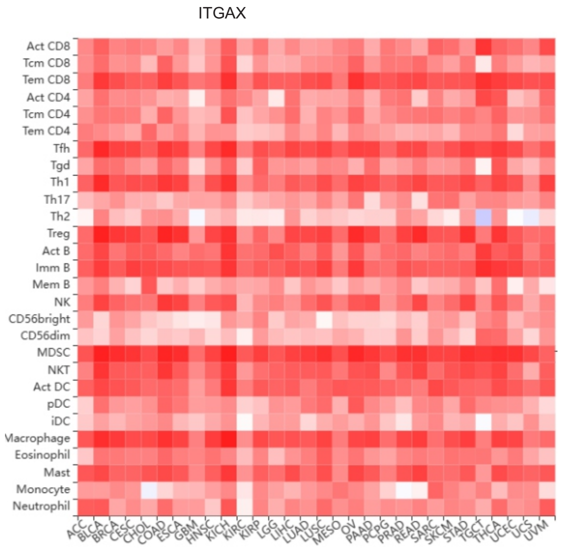

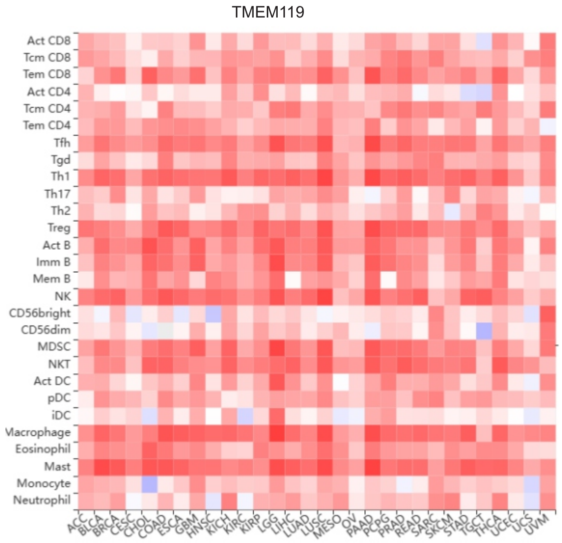

Figure S7. Correlation of ITGAL, ITGAX, and TMEM119 with cancer-associated immune cells. Spearman correlations of ITGAL, ITGAX, and TMEM119 with the indicated immune cell types across n = 30 human cancer types. Correlations were determined using TISIDB.

Figure S8

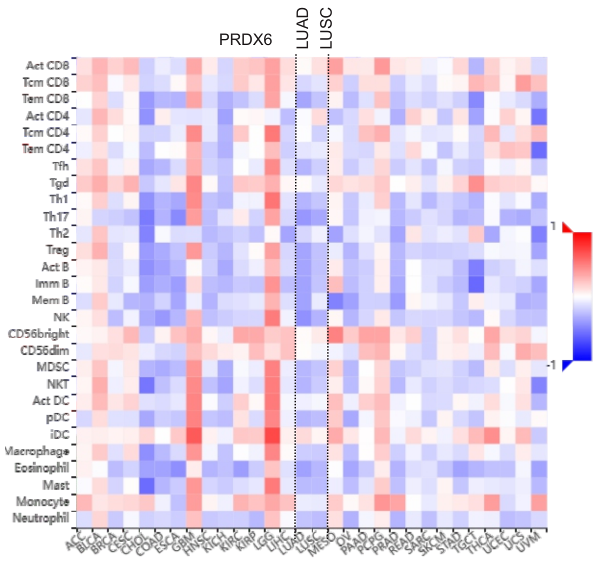

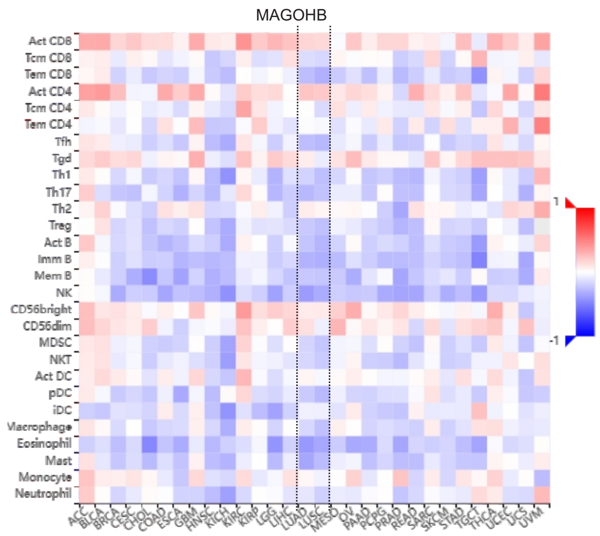

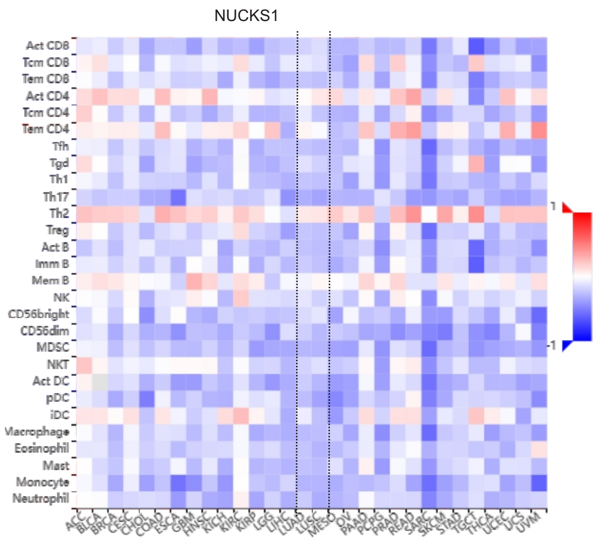

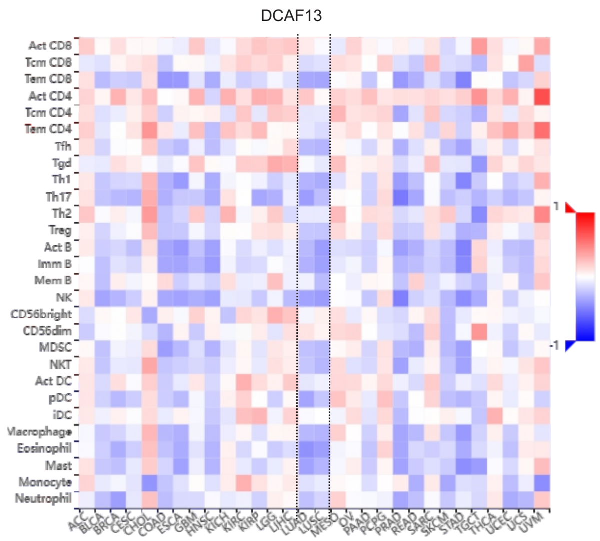

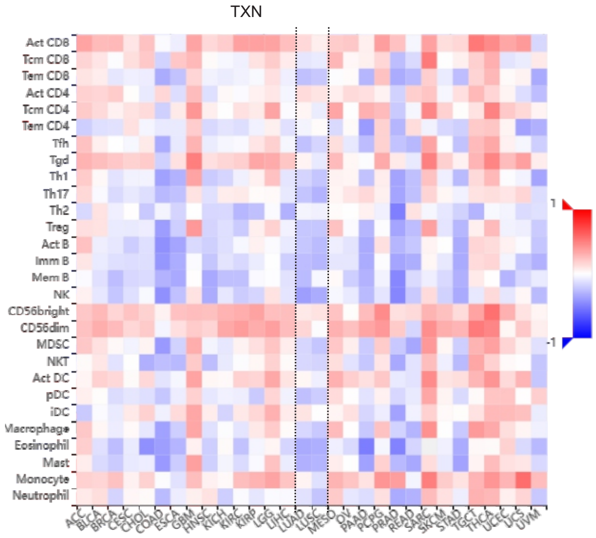

Figure S8. Association of taxifolin-upregulated genes with immune cell content in cancers. PRDX6, MAGOHB, NUCKS1, DCAF13, and TXN, whose mouse homologous genes were upregulated by taxifolin in LL2 tumors, were analyzed for spearman correlation with the indicated immune cell types across 30 human cancer types using the TISIDB platform. Both LUAD and LUSC cancer types are bordered by two dotted lines. Please note the general negative correlation of these genes with the indicated immune checkpoints in LUAD and LUSC.

Figure S9

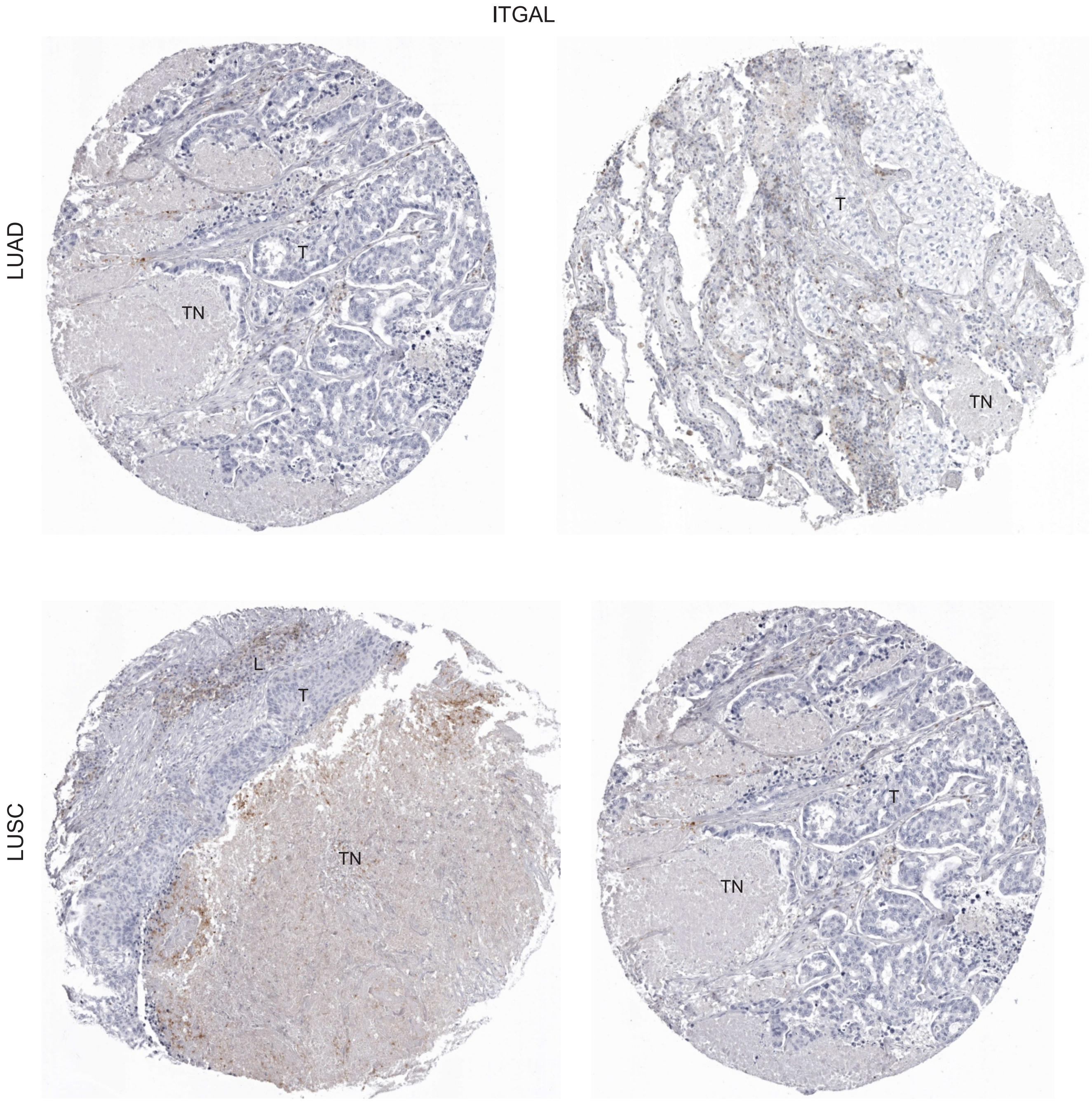

Figure S9. ITGAL protein expression in LUAD and LUSC. Two images each for IHC staining of ITGAL in LUAD and LUSC tissue microarray were downloaded from the Human Protein Atlas (https://www.proteinatlas.org/). T: tumor cells; TN: tumor necrosis.

Figure S10

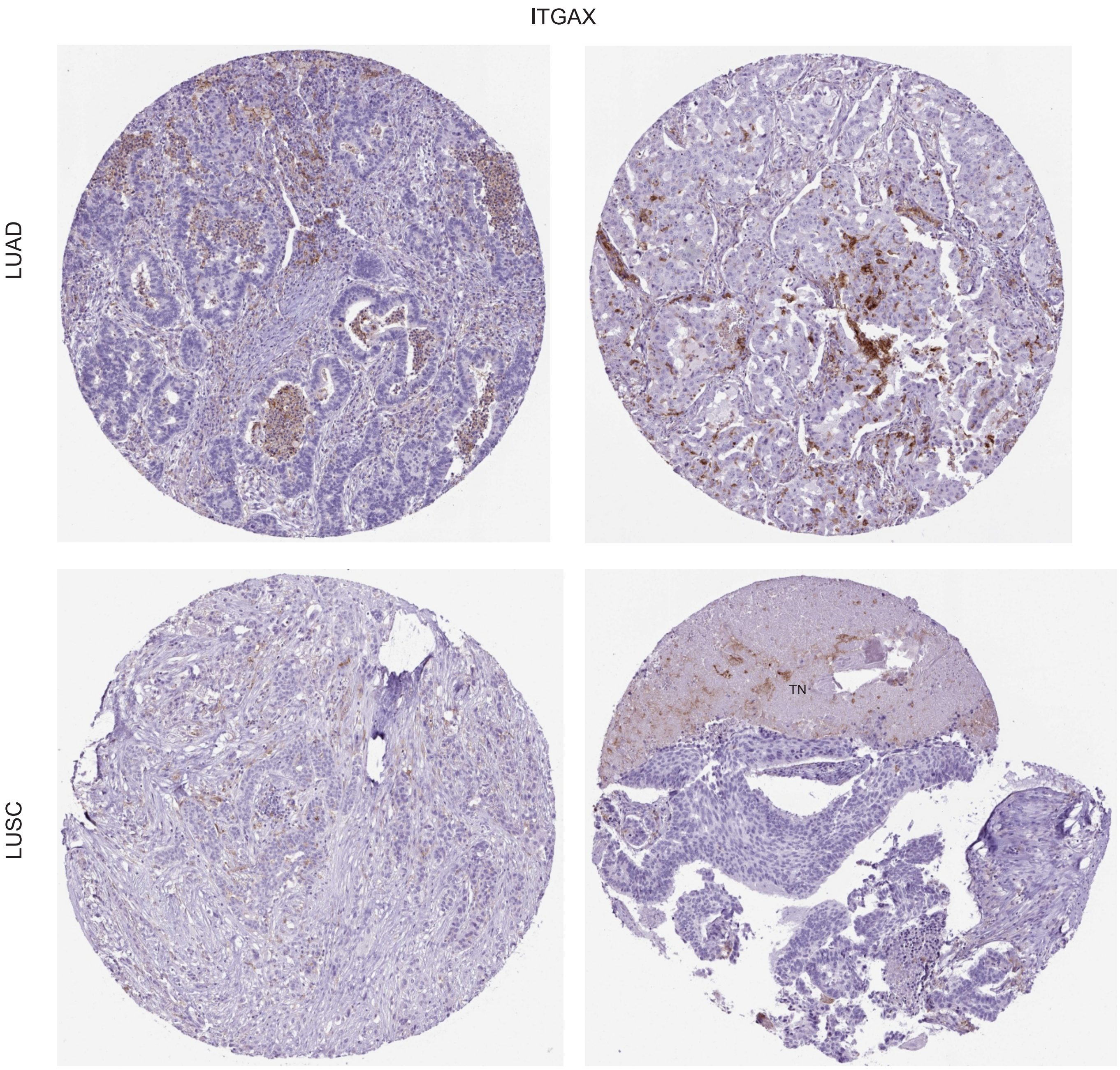

Figure S10. ITGAX protein expression in LUAD and LUSC. Two images each for IHC staining of ITGAX in LUAD and LUSC tissue microarray were downloaded from the Human Protein Atlas (https://www.proteinatlas.org/). TN: tumor necrosis.

Figure S11

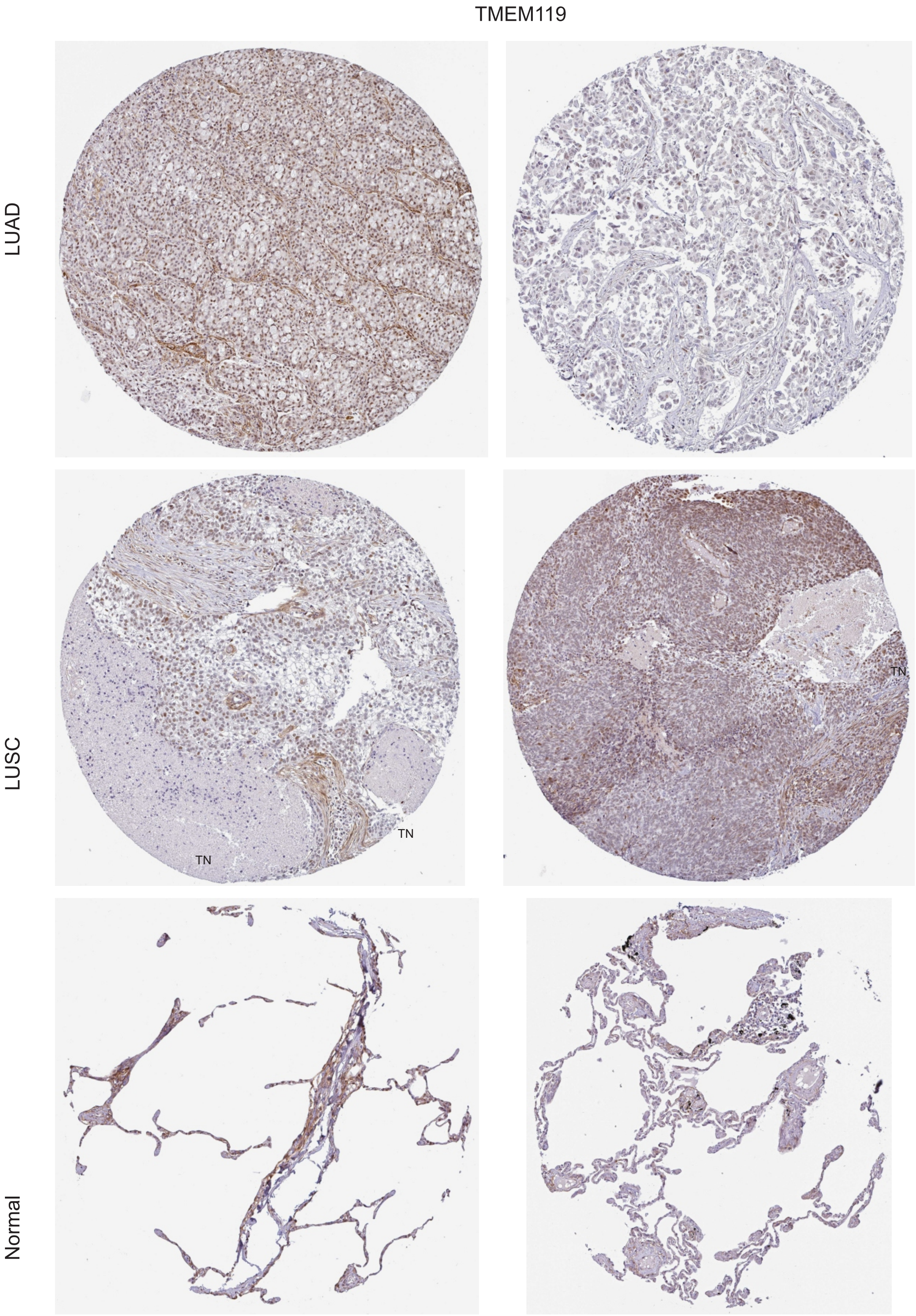

Figure S11. TMEM119 protein expression in LUAD and LUSC. Two images each for IHC staining of TMEM119 in LUAD and LUSC tissue microarray were downloaded from the Human Protein Atlas (https://www.proteinatlas.org/). T: tumor cells; TN: tumor necrosis. Two staining of TMEM119 in normal tissues are also included.

Figure S12

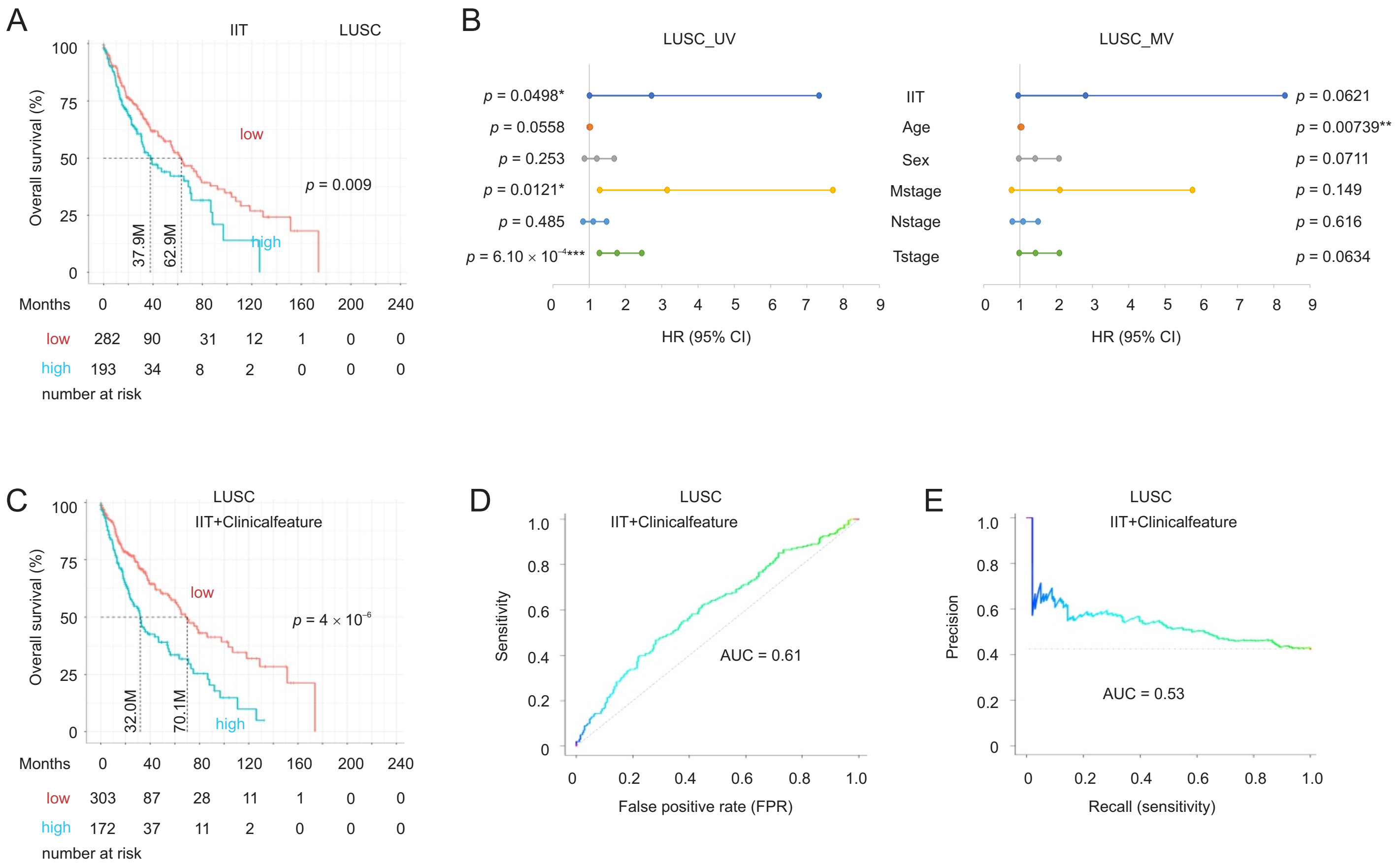

Figure S12. Prediction of OS by IIT in LUSC. (A) Stratification of mortality risk of LUSC using the TCGA PanCancer Atlas LUSC dataset. (B) Univariate (UV) and multivariate (MV) Cox analysis of LUAD mortality risk using IIT and the indicated clinical features. Sex: men compared to women; Mstage: M1 versus M0; Nstage: 1 (N ≥ 1) versus N0; and Tstage: 1 (T3+T4) compared to 0 (T1+T2). (C) Stratification of mortality risk by IIT together with the above clinical features (IIT+Clinicalfeature). (D, E) Discrimination of OS by IIT+Clinicalfeature with ROC-AUC (receiver operating characteristic-area under the curve) and precision recall (PR)-AUC curves for LUSC.
