# Supplementary material for: Taxifolin Inhibits the Growth of Non-Small-Cell Lung Cancer via Downregulating Genes Displaying Novel and Robust Associations with Immune Evasion Factors
Source: Cancers (Basel). 2023 Sep 30;15(19):4818. doi: 10.3390/cancers15194818 (PMC10571863; doi:10.3390/cancers15194818)
Supplement: Supplementary file 1 [file cancers-15-04818-s001.zip › Table S3.pdf]

Table S3. Spearman correlations among ITGAL, ITGAX, and TMEM119 in LUAD and LUSC

	ITGAL	ITGAX	TMEM119
ITGAL	1	rho = 0.76, *p* = 2.88e-96[i]<br>rho = 0.79, *p* = 2.90e-107[ii]	rho = 0.50, *p* = 6.13e-33[i]<br>rho = 0.66, *p* = 1.90e-64[ii]
ITGAX		1	rho = 0.40, *p* = 1.08e-20[i]<br>rho = 0.64, *p* = 1.82e-58[ii]
TMEM119			1

Spearman correlation (rho) was determined using the TCGA Firehole Legacy LUAD (n = 510) and LUSC (n = 501) datasets; i: LUAD; ii: LUSC
